# Are U‐shaped relationships between risk factors and outcomes artifactual?

**DOI:** 10.1111/1753-0407.13335

**Published:** 2022-12-08

**Authors:** Reema Shah, Lehana Thabane, Hertzel C. Gerstein

**Affiliations:** ^1^ Population Health Research Institute, Hamilton Health Sciences and McMaster University Hamilton Ontario Canada

**Keywords:** cardiovascular outcome, insulin, J shape, risk factor, systolic blood pressure, U‐shape, weight, U型, J型, 风险因素, 体重, 收缩压, 胰岛素, 心血管结局

## Abstract

**Background:**

The objective of this study was to evaluate whether the observed nadir in a U‐ or J‐shaped relationship between a particular risk factor and a future health outcome is a function of the distribution of the risk factor in the sample being analyzed.

**Methods:**

Data from the ORIGIN trial were used to assess the relationship between three risk factors (weight, systolic blood pressure, and serum insulin) and the hazard of a major cardiovascular event comprising a nonfatal myocardial infarction, nonfatal stroke, or cardiovascular death. Three spline curves were generated for each risk factor. The first was based on all available data, the second for a subgroup with a higher mean risk factor level, and the third for a subgroup with a lower mean risk factor level. Nadir levels of the risk factor (i.e., risk factor levels predicting the lowest hazard) were then identified for each spline curve.

**Results:**

When compared to the nadir values based on all available data, nadir values for all three risk factors were higher for the subgroups with higher mean levels and lower for those with lower mean levels.

**Conclusions:**

The distribution of a risk factor in the population is an important determinant of its nadir value. Populations with high or low values may have high and low nadirs, respectively. Identification of a nadir for a modifiable risk factor from epidemiologic relationships may therefore arise from this distribution bias and is therefore unrelated to therapeutic targets.

## Graphical abstract

**Highlights**

- U‐shaped relationships between three common cardiovascular risk factors (weight, systolic blood pressure, and insulin) and hazards of composite cardiovascular outcomes were analyzed in three study samples: entire sample, “high” subgroup, and “low” subgroup.
- Nadir values for all three risk factors were shifted left in the low‐subgroup population and shifted right in the high‐subgroup population when compared to the nadir value in the original population.
- Recommendations that risk factor levels below or above some specific nadir should not be therapeutically targeted in the underlying population based on U‐ or J‐ shape relationships observed in epidemiologic studies should be supported by randomized controlled trials.

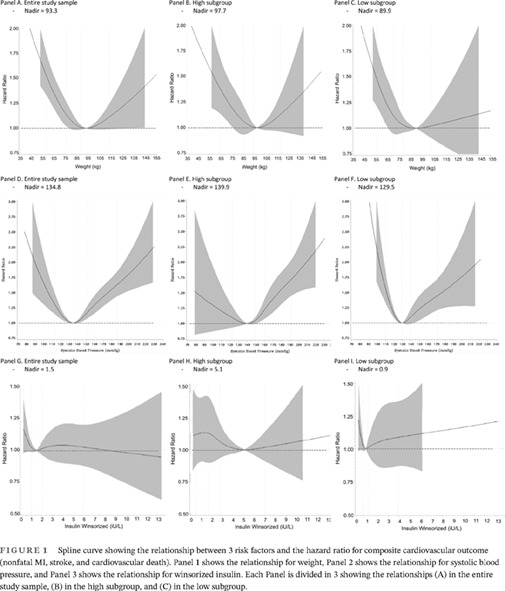

## 摘要

目的:评估某一特定危险因素与未来健康结果之间的U型或J型关系中观察到的最低点是否为被分析样本中风险因素分布的函数。

研究设计和设置:ORIGIN试验的数据用于评估3个危险因素(体重、收缩压和血清胰岛素)与主要心血管事件(MACE)风险之间的关系, 包括非致死性心肌梗死、非致死性卒中或心血管死亡。每个危险因素生成三条样条曲线。第一项研究基于所有可用数据, 第二项研究针对平均风险因素水平较高的亚组, 第三项研究针对平均风险因素水平较低的亚组。然后为每条样条曲线确定风险因素的最低水平(即预测最低风险的风险因素水平)。

结果:与所有可用数据的最低点相比, 所有3个危险因素的最低点在平均水平较高的亚组较高, 在平均水平较低的亚组较低。

结论:危险因素在人群中的分布是其最低点值的重要决定因素。具有高或低值的种群可能分别具有高和低的最低点。因此, 从流行病学关系中确定可改变风险因素的最低点可能源于这种分布偏差, 因此与治疗靶点无关。

## 1 INTRODUCTION

Many epidemiologic studies have reported a U‐ or J‐shaped relationship between a variety of risk factors and future serious health outcomes, in which both high and low levels of the risk factor are more strongly related to the outcome than intermediate levels of the risk factor.^1^, ^2^ Examples of such relationships include the link between systolic blood pressure (BP), diastolic BP,^3^, ^4^, ^5^, ^6^ fasting blood glucose,^7^ glycosylated hemoglobin (HbA1c),^8^ body mass index,^9^, ^10^ sodium intake,^11^ alcohol intake,^12^ and 25‐OH vitamin D^13^ with mortality; low‐density lipoprotein cholesterol levels,^14^ BP,^5^ and sodium intake^15^ with cardiovascular events; and birthweight with stillbirth.^16^

These epidemiologic observations have been used to justify the conclusion that levels of a certain risk factor that are below or above a specific nadir are unsafe, and if that risk factor is modifiable, those lower or higher levels should be avoided. However, different nadirs for the relationship between a specific risk factor and an outcome have been reported in different studies. Thus, for BP, the nadir has ranged from 110 to 169 mm Hg and from 55 to 94 mm Hg for systolic and diastolic BP, respectively.^2^ These differences may be due to biases such as reverse causation, confounding by other residual risk factors such as critical illness or selection bias, and low numbers of individuals with either low or high levels of the risk factor.^2^, ^17^, ^18^, ^19^ An additional, novel explanation for these relationships is the possibility that the observed nadir for the relationship between a particular risk factor and future health outcome is related to the distribution of the risk factor within the population sample being analyzed.

The ORIGIN trial^20^, ^21^, ^22^ recruited and followed 12 537 participants with dysglycemia and other cardiovascular risk factors for a median of 6.2 years during which the effect of insulin glargine and/or omega 3 fatty acids on cardiovascular and other outcomes was assessed. ORIGIN trial data were used to identify the levels of three common cardiovascular risk factors (weight, systolic BP, and plasma insulin) at baseline that predicted the lowest incidence of the composite cardiovascular outcome (i.e., the nadir value) and to test the hypothesis that these nadirs emerge as a consequence of the baseline distribution of the risk factor within the population.

## 2 METHODS

### 2.1 Study design and sample studied

The design, baseline characteristics, and results of the ORIGIN trial have been previously published.^20^ The trial was approved by each site's institutional review boards, and all participants provided written informed consent. In addition, a subset of 8401 participants consented to the collection, storage, and analysis of blood samples. The epidemiologic analyses described here were restricted to individuals for whom baseline systolic BP, weight, or insulin levels were available.

### 2.2 Insulin measurement

As outlined in the ORIGIN biomarker study,^23^ serum insulin levels were measured by Myriad RBM Inc. as part of a customized, extended version of the company's Human Discovery Multi‐Analyte Profile (MAP) 250+ panel on the LUMINEX 100/200 platform biomarker panel in 1 ml of stored serum.^23^ Baseline serum insulin was further winsorized^24^ to exclude outliers beyond four standard deviations.

### 2.3 Outcome

The primary outcome for these analyses was the first occurrence of a major cardiovascular event (MACE) defined as either a nonfatal myocardial infarction, nonfatal stroke, or cardiovascular death based on masked adjudication during the conduct of the study.

### 2.4 Relationship between weight, systolic BP, serum insulin, and cardiovascular outcome

The effect of the distribution of these three risk factors within the ORIGIN sample on their respective nadir values was analyzed as follows. First, spline curves modeling the relationship between each risk factor and the hazard of MACE were constructed using a restricted cubic spline function with four knots.^25^, ^26^ Second, a high‐distribution subgroup was created by randomly selecting 80% of people whose risk factor level was at or above the median value of this risk factor and 20% of people whose risk factor level was below the median. Third, a low‐distribution subgroup was created by randomly selecting 80% of people whose risk factor level was less than the median value of this risk factor and 20% of people whose risk factor level was at or above the median. Finally, similar spline curves were generated for the high‐ and low‐distribution subgroups and were used to identify high‐ and low‐distribution nadirs by inspection. Those with missing values of the risk factor of interest were excluded.

### 2.5 Statistical analysis

Cox proportional hazard models adjusted for age, sex, baseline diabetes status, and ethnicity were used to evaluate the relationship between risk factors and MACE. As the focus of these analyses was on univariate relationships, no further adjustments were done. SAS version 9.4 (SAS Institute Inc.) was used for all analyses.

## 3 RESULTS

As noted in Table 1, 12 527 participants were included in the weight analyses, 12 525 in the systolic BP analyses, and 8401 participants for whom insulin levels were available were included in the insulin analyses. Participants for all three analyses had a mean age of 64 years, 59% had a prior cardiovascular event, and 12% had prediabetes. Their mean weight varied between 83 and 84 kg, mean systolic BP was 146 mm Hg, and mean serum insulin level was 2.0 IU/L.

**TABLE 1:** Baseline characteristics for entire population included for the systolic blood pressure (SBP), weight, and insulin analyses

	Weight	SBP	Serum insulin
*N*	12 527	12 525	8401
Age (years) (SD)	63.5 (7.8)	63.5 (7.8)	63.7 (7.9)
Female sex (%)	4384 (35.0)	4380 (35.0)	2848 (33.9)
Prior CV event (%)	7372 (58.9)	7373 (58.9)	4991 (59.4)
Overt diabetes (%)	11 071 (88.4)	11 070 (88.4)	7390 (88.0)
Prediabetes (%)	1452 (11.6)	1451 (11.6)	1008 (12.0)
Hypertension %)	9957 (79.5)	9955 (79.5)	6638 (79.0)
Dyslipidemia (%)	8234 (65.7)	8230 (65.7)	5441 (64.8)
Current smoker (%)	1551 (12.4)	1551 (12.4)	1050 (12.5)
Baseline DBP (mm Hg) (SD)	84.1 (12.1)	84.1 (12.0)	84.4 (12.1)
Baseline HbA1C (%) (SD)	6.5 (0.9)	6.5 (0.9)	6.5 (1.0)
Baseline fasting BG (mmol/L) (SD)	7.3 (2.0)	7.3 (2.0)	7.3 (2.0)
Baseline LDL (mmol/L) (SD)	2.9 (1.0)	2.9 (1.0)	2.9 (1.0)
Baseline creatinine (mmol/L) (SD)	89.0 (22.0)	89.0 (22.0)	89.3 (22.2)
Weight (kg) (SD)	83.2 (17.0)	83.2 (17.0)	84.4 (17.7)
Baseline SBP (mm Hg) (SD)	145.8 (21.8)	145.8 (21.8)	146.3 (21.8)
Baseline insulin (IU/L) (SD)	2.0 (2.1)	2.0 (2.1)	2.0 (2.1)

*Note*: All patients with missing data for that risk factor were excluded.

Abbreviations: BG, blood glucose; CV, cardiovascular; DBP, diastolic blood pressure; HbA1c, glycosylated hemoglobin; LDL, low‐density lipoprotein.

As expected, participants included in the high risk factor level subgroup had higher mean levels of the risk than those in the low risk factor level subgroup factor, whereas the mean level of both subgroups combined fell between the two subgroups. For example, the mean (SD) weights of 6312 and 6217 participants in the high‐ and low‐level groups were 91.1 kg (15.8) and 75.3 kg (14.4), respectively, whereas the mean weight of all 12 527 participants was 83.2 kg (17.0) (Table 2). Similar patterns were noted for systolic BP (Table 3) and serum insulin concentrations (Table 4). Mean levels or proportions of other variables in participants assigned to the two subgroups for each of the three risk factors are noted in Tables 2, 3, 4.

**TABLE 2:** Baseline characteristics and weight data for the entire population, higher‐weight subgroup, and lower‐weight subgroup for the weight analyses

	All participants	Higher weight	Lower weight
*N*	12 527	6312	6217
Age (years) (SD)	63.5 (7.8)	62.7 (7.6)	64.3 (8.0)
Female sex (%)	4384 (35.0)	1807 (28.6)	2578 (41.5)
Prior CV event (%)	7372 (58.9)	3776 (59.8)	3627 (58.3)
Overt diabetes (%)	11 071 (88.4)	5559 (88.1)	5518 (88.8)
Prediabetes (%)	1452 (11.6)	751 (11.9)	697 (11.2)
Hypertension (%)	9957 (79.5)	5090 (80.6)	4857 (78.1)
Dyslipidemia (%)	8234 (65.7)	4270 (67.7)	3958 (63.7)
Current smoker (%)	1551 (12.4)	828 (13.1)	726 (11.7)
Baseline SBP (mm Hg) (SD)	145.8 (21.8)	145.8 (21.2)	145.7 (22.5)
Baseline DBP (mm Hg) (SD)	84.1 (12.1)	85.1 (12.0)	83.2 (12.1)
Baseline HbA1c (%) (SD)	6.5 (0.9)	6.5 (0.9)	6.5 (1.0)
Baseline fasting BG (mmol/L) (SD)	7.3 (2.0)	7.4 (1.9)	7.3 (2.0)
Baseline insulin (IU/L) (SD)	2.0 (2.1)	2.3 (2.1)	1.8 (1.9)
Baseline LDL (mmol/L) (SD)	2.9 (1.0)	2.9 (1.0)	2.9 (1.0)
Baseline creatinine (mmol/L) (SD)	89.0 (22.0)	89.6 (21.6)	88.6 (22.4)
CV outcome (%)	2053 (16.4%)	967 (15.3%)	1063 (17.1%)
Nadir weight (kg)	93.3	97.7	89.9
Mean weight (kg) (SD)	83.2 (17.0)	91.1 (15.8)	75.3 (14.4)
Median weight (Q1, Q3)	82.0 (71.7, 93.3)	90.0 (83.0, 100.0)	74.1 (66.3, 80.5)

*Note*: All patients with missing data for weight were excluded.

Abbreviations: BG, blood glucose; CV, cardiovascular; DBP, diastolic blood pressure; HbA1c, glycosylated hemoglobin; LDL, low‐density lipoprotein; SBP, systolic blood pressure.

**TABLE 3:** Baseline characteristics and systolic blood pressure (SBP) data for the entire population, higher‐SBP subgroup, and lower‐SBP subgroup for the SBP analyses.

	All participants	Higher SBP	Lower SBP
*N*	12 525	6304	6223
Age (years) (SD)	63.5 (7.8)	64.1 (7.8)	62.9 (7.8)
Female sex (%)	4380 (35.0)	2312 (36.7)	2080 (33.4)
Prior CV event (%)	7373 (58.9)	3507 (55.6)	3865 (62.1)
Overt diabetes (%)	11 070 (88.4)	5620 (89.2)	5447 (87.5)
Prediabetes (%)	1451 (11.6)	682 (10.8)	773 (12.4)
Hypertension	9955 (79.5)	5362 (85.1)	4600 (73.9)
Dyslipidemia (%)	8230 (65.7)	4102 (65.1)	4102 (65.9)
Current smoker (%)	1551 (12.4)	721 (11.4)	845 (13.6)
Weight (kg) (SD)	83.2 (17.0)	83.2 (17.1)	83.2 (17.1)
Baseline DBP (mm Hg) (SD)	84.1 (12.0)	88.0 (12.0)	80.2 (10.8)
Baseline HbA1c (%)	6.5 (0.9)	6.5 (1.0)	6.5 (0.9)
Baseline fasting BG (mmol/L) (SD)	7.3 (2.0)	7.4 (2.0)	7.2 (2.0)
Baseline insulin (IU/L) (SD)	2.0 (2.1)	2.0 (2.0)	2.1 (2.1)
Baseline LDL (mmol/L) (SD)	2.9 (1.0)	3.0 (1.0)	2.8 (1.0)
Baseline creatinine (mmol/L) (SD)	89.0 (22.0)	89.0 (22.4)	89.0 (21.5)
CV outcome (%)	2053 (16.4%)	1098 (17.4%)	954 (15.3%)
Nadir SBP (mm Hg)	134.8	139.9	129.5
Mean SBP (mm Hg) (SD)	145.8 (21.8)	156.0 (20.1)	135.4 (18.1)
Median SBP (Q1, Q3)	143.5 (130.5, 159.0)	154.5 (145.0, 167.0)	134.0 (124.5, 142.0)

*Note*: All patients with missing data for SBP were excluded.

Abbreviations: BG, blood glucose; CV, cardiovascular; DBP, diastolic blood pressure; HbA1c, glycosylated hemoglobin; LDL, low‐density lipoprotein.

**TABLE 4:** Baseline characteristics and insulin data for the entire population, higher‐insulin subgroup, and lower‐insulin subgroup for the insulin analyses

	All participants	Higher insulin	Lower insulin
*N*	8401	4333	4070
Age (years) (SD)	63.7 (7.9)	63.1 (7.8)	64.3 (8.0)
Female sex (%)	2848 (33.9)	1519 (35.1)	1322 (32.5)
Prior CV event (%)	4991 (59.4)	2607 (60.2)	2418 (59.4)
Overt diabetes	7390 (88.0)	3821 (88.2)	3576 (87.9)
Prediabetes (%)	1008 (12.0)	511 (11.8)	491 (12.1)
Hypertension (%)	6638 (79.0)	3455 (79.7)	3195 (78.5)
Dyslipidemia	5441 (64.8)	2915 (67.3)	2561 (62.9)
Current smoker (%)	1050 (12.5)	549 (12.7)	489 (12.0)
Weight (kg) (SD)	84.4 (17.7)	87.5 (17.4)	81.2 (17.3)
Baseline SBP (mm Hg) (SD)	146.3 (21.8)	146.0 (21.2)	146.5 (22.4)
Baseline DBP (mm Hg) (SD)	84.4 (12.1)	84.7 (12.0)	84.0 (12.1)
Baseline HbA1c (%) (SD)	6.5 (1.0)	6.5 (1.0)	6.5 (1.0)
Baseline fasting BG (mmol/L) (SD)	7.3 (2.0)	7.4 (2.0)	7.2 (2.0)
Baseline LDL (mmol/L) (SD)	2.9 (1.0)	2.9 (1.0)	2.9 (1.0)
Baseline creatinine (mmol/L) (SD)	89.3 (22.2)	89.5 (22.0)	89.6 (22.7)
CV outcome (%)	840 (16.7%)	708 (16.3%)	704 (16.7%)
Nadir insulin (IU/L)	1.5	5.1	0.9
Mean insulin (IU/L) (SD)	2.0 (2.1)	2.8 (2.2)	1.2 (1.4)
Median insulin (Q1, Q3)	1.4 (0.7, 2.6)	2.2 (1.5, 3.3)	0.9 (0.5, 1.3)

*Note*: All patients with missing data for insulin were excluded.

Abbreviations: BG, blood glucose; CV, cardiovascular; DBP, diastolic blood pressure; HbA1c, glycosylated hemoglobin; LDL, low‐density lipoprotein; SBP, systolic blood pressure.

**FIGURE 1:**
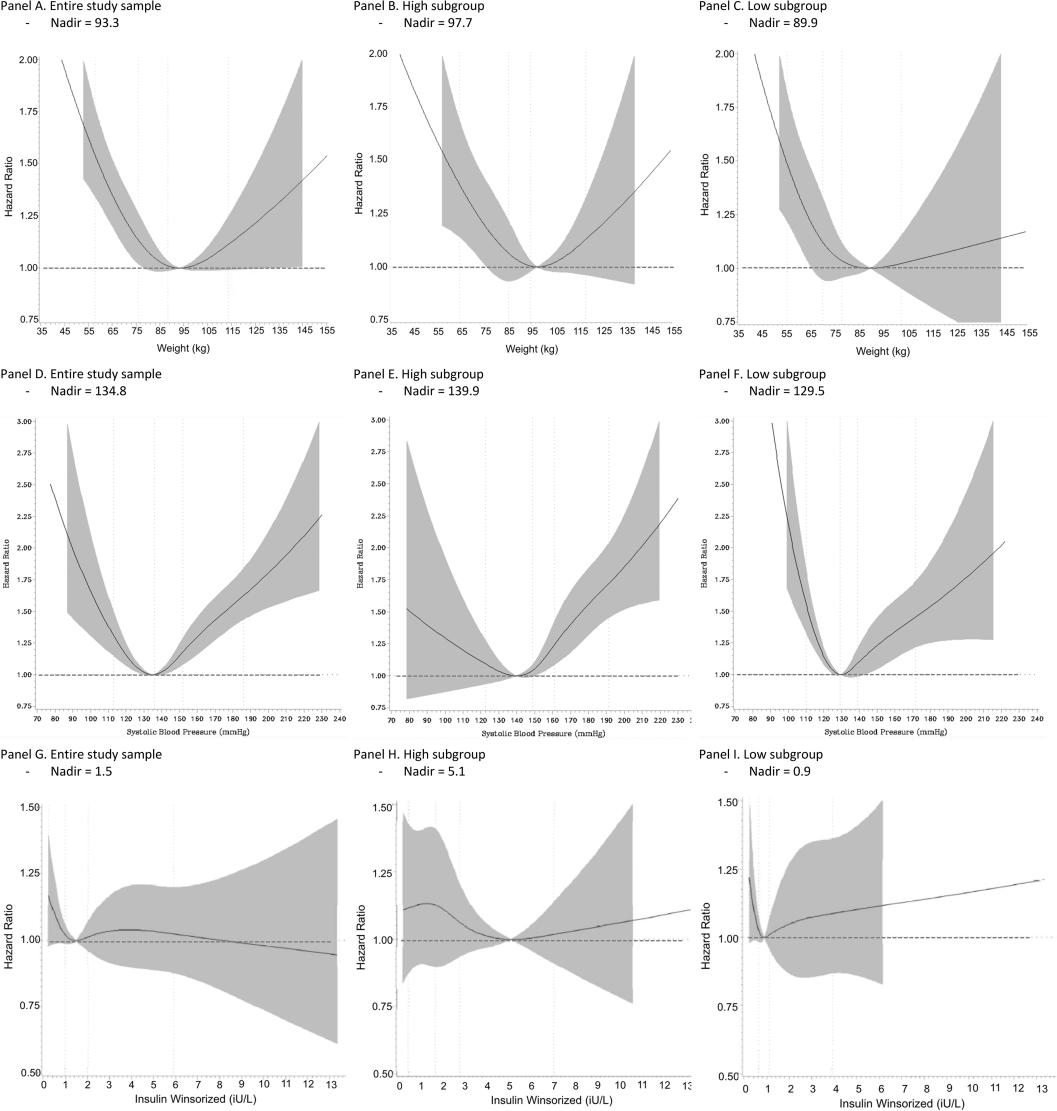
Spline curve showing the relationship between 3 risk factors and the hazard ratio for composite cardiovascular outcome (nonfatal MI, stroke, and cardiovascular death). Panel 1 shows the relationship for weight, Panel 2 shows the relationship for systolic blood pressure, and Panel 3 shows the relationship for winsorized insulin. Each Panel is divided in 3 showing the relationships (A) in the entire study sample, (B) in the high subgroup, and (C) in the low subgroup.

Spline curves for the relationship between each of the three risk factors (weight, systolic BP, and serum insulin) and MACE that were based on all available data (i.e., combined subgroups) were all consistent with a U‐ or J‐shaped relationship (Figure 1A,D,G). Notably, for each of the three risk factors, the relationship between the risk factor and MACE in the high‐level subgroup was distributed around a higher nadir level of the risk factor than in the low‐level subgroup, whereas the nadir of the of the combined subgroups fell between the two (Figure 1B,C,E,F,H,I). Thus, the nadirs for the high‐weight, low‐weight, and combined subgroups were 97.7, 89.9, and 93.3 kg, respectively; for systolic BP, 139.9, 129.5, and 134.8 mm Hg, respectively; and for serum insulin concentrations, 5.12, 0.9, and 1.47 IU/L, respectively.

## 4 DISCUSSION

This epidemiologic analysis of more than 12 000 people with a baseline weight and BP and more than 8000 people with a baseline insulin level demonstrated a U‐ or J‐shaped relationship between each of these measurements and incident cardiovascular events occurring during a median follow‐up period of 6.2 years. Our study adds to the existing body of literature that has shown a U‐shaped relationship between BP,^3^, ^5^, ^6^ weight,^27^ and insulin^28^, ^29^, ^30^ and cardiovascular events. It also demonstrates that the nadir value for each of these risk factors was determined by the mean value of the risk factor in the sample. Samples with a higher mean level had a higher nadir, and samples with a lower mean had a lower nadir. Thus, at least for these three risk factors, the distribution of the risk factor within the sample analyzed is an important determinant of the nadir of the relationship.

These findings have a variety of implications. First, when a U‐ or J‐shaped relationship is reported, investigators should also report the distribution of that risk factor within the population. Second, it also demonstrates that the nadir value of the U for each of these risk factors is affected by the distribution of the risk factor in the population such that populations with a higher mean or median value had a higher nadir and populations with a lower mean or median value had a lower nadir. Third, identification of a nadir for a modifiable risk factor (e.g., BP) from epidemiologic relationships should be construed as hypothesis generating with respect to therapeutic targets, and the therapeutic implications of targeting a particular value need to be explored in randomized controlled trials. For example, BP researchers have clearly shown that therapies that drive the systolic BP below the epidemiologic nadir clearly reduce outcomes.^31^

The sample size, number of outcomes, and qualitative consistency of findings for three different risk factors are clear strengths of these analyses. The fact that the distributions of the three risk factors were all drawn from the same population limits generalizability while increasing confidence that the findings are not due to unmeasured determinants of the distribution. Although these analyses do not imply that when there is a U‐ or J‐shaped relationship between risk factors and outcomes, the nadir is always related to its distribution within the population, they illustrate that this possibility must be considered before ascribing biologic or therapeutic significance to the distribution. Simulation studies would be required to determine whether this phenomenon holds true for all U‐shaped relationships between risk factors and health outcomes.

In conclusion, the distribution of a risk factor in the population is an important determinant of its nadir value, and populations with high or low values may have high and low nadirs, respectively. The possibility of a distribution bias should therefore always be considered when confronted with a U‐ or J‐shaped relationship between a risk factor and an outcome.

## AUTHOR CONTRIBUTIONS

Reema Shah: Study design; statistical analysis; interpretation of results; drafting the manuscript; guarantor. Lehana Thabane: Study concept and design; critical revision of the manuscript. Hertzel C. Gerstein: Study concept and design; interpretation of results; drafting and critical revision of the manuscript.

## FUNDING INFORMATION

The ORIGIN trial was funded by Sanofi.

## DISCLOSURE STATEMENT

Reema Shah and Lehana Thabane have no conflicts of interest to disclose. Hertzel C. Gerstein has received research grant support from Sanofi, Lilly, AstraZeneca, and Merck and honoraria for speaking or consulting from Sanofi, Novo Nordisk, Lilly, Boehringer Ingelheim, AstraZeneca, Merck, and Abbot.
